# Rates and trends in cesarean sections between 2008 and 2012 in Iraq

**DOI:** 10.1186/s12884-016-1211-6

**Published:** 2017-01-11

**Authors:** Nazar P. Shabila

**Affiliations:** Department of Community Medicine, College of Medicine, Hawler Medical University, Erbil, Iraq

## Abstract

**Background:**

The prevalence of cesarean sections is increasing globally; however, it can lead to significant increases in maternal and infant morbidity and mortality. This study aimed to determine the rates and trends of cesarean sections in Iraq.

**Methods:**

The cesarean section rates of all births and public and private hospital-based births were calculated from the data on births provided by the annual reports of the Iraqi Ministry of Health for the years 2008 and 2012. The comparable rates for the Center/South and Kurdistan Region and the individual governorates were determined. The cesarean section rates for all births in 2008 were computed and compared with the 2012 rates.

**Results:**

The cesarean section rate for all births in Iraq was 24.4% in 2012, which was similar to the rates in the Iraqi Kurdistan Region (25.4%) and the Center/South of Iraq (24.3%). The cesarean section rates were specifically high in the governorates containing a larger number of private hospitals, and there was a significant positive relationship between the number of private hospitals and the cesarean section rate (beta = 0.671; *r* = 0.671; *P* < 0.002). The hospital-based cesarean section rate was 34.7%. The cesarean section rate in private hospitals (77.9%) was remarkably higher than the rate in public hospitals (29.3%). The overall rate of cesarean sections in Iraq increased from 18.0% in 2008 to 24.4% in 2012.

**Conclusions:**

The cesarean section rate in Iraq is far above the recommended rate. Iraq witnessed a rapid upward trend in the cesarean section rate from 2008 to 2012, with most of this trend attributable to the Kurdistan Region. There is a potential relationship between the expansion of the private health sector and the increasing cesarean section rate, and further studies of this relationship are necessary. Future research should consider an audit of the indications for a cesarean section rather than measuring the cesarean section rate alone.

## Background

Cesarean sections are a commonly performed operation for women. The procedure is associated with significant increases in maternal and infant morbidity and mortality, particularly in low-income countries. Increases in maternal morbidity are particularly prevalent after an emergency cesarean section or a cesarean section performed during the second stage of labor [1–3].

The prevalence of cesarean sections is increasing globally each year where the rates have increased beyond the recommended level of 10%. Cesarean section rates beyond this level do not further reduce maternal and perinatal mortality [4, 5]. There is considerable variation in the rates of cesarean sections, particularly between high- and low-income countries and between different institutions within these countries [1, 6]. Many factors have contributed to the increasing rates of cesarean sections including medical and non-medical factors. Examples of medical factors include increases in maternal age and body mass index and changes in obstetric practices and technologies. Examples of non-medical factors include cesarean sections requested by the mother, fear of litigation among caregivers, the inappropriate organization of maternity care and physician-induced demand for cesarean sections [7, 8].

There is significant variation in the cesarean section rate in terms of socioeconomic status. However, research has suggested that the increasing rate is attributed to structural factors related to service supply and compensation structure and not a woman’s ability to pay [9]. A systematic review and meta-analysis of observational studies revealed that the preference for a cesarean section in women is greater than 15% [10]. The same review concluded that the actual contribution of women demanding the procedure to the rising cesarean section rates is not well studied. Recent evidence also shows that the demand for a cesarean section among young, educated women residing in urban areas has increased [11].

According to the limited available research, the cesarean section rate in Iraq is considerably higher than the recommended upper limit of 10% [12, 13]. Research concerning the factors contributing to the increased rate of cesarean sections in Iraq is very scarce. The private healthcare sector in Iraq has witnessed a rapid unguided expansion during the last decade [14]. The poor governmental oversight and regulation of this sector have raised concerns about a possible increase in the physician-induced demand for healthcare including cesarean sections [15–17]. The aim of this study was to determine the rates and trends of cesarean sections in Iraq. This study also sought to determine any differences in the cesarean section rates according to the governorates and regions as well as the size of the private healthcare sector.

## Methods

This descriptive study is based on the data on births provided by the Iraqi Ministry of Health. The data on total births, public and private hospital-based births and births by cesarean section were extracted from the 2012 Annual Report of the Iraqi Ministry of Health, which is the latest published report. The report, which is publicly available on the ministry’s website, is primarily a compilation of institutional and administrative records submitted by the directorates of health of the Iraqi governorates [18]. The annual report of the Ministry of Health contains summary tables that include the data on all births registered during the reporting year distributed by the place of occurrence such as homes, public hospitals or private hospitals. These data are also segregated at the governorate levels. The data from the annual report is considered adequately reliable because birth registration in Iraq was almost universal in 2006 and 2011. The Multiple Indicator Cluster Survey (MICS) 3 and 4 showed that the completeness of birth registration in Iraq was 95% in 2006 [19] and 99.2% in 2011 [20]. In 2012, there were no specific areas in Iraq outside of the control of the government that might have lacked proper registration of births. This study was approved by the Research Ethics Committee of Hawler Medical University, and authorization of the Ministry of Health was obtained to use the data from the annual reports.

The cesarean section rates for all births and hospital-based births were calculated for each governorate. The comparable rates for the Center/South of Iraq and Iraqi Kurdistan Region were determined. The segregated rates of public hospitals and private hospitals were also calculated for each governorate with the exception of the three governorates of the Iraqi Kurdistan Region because these segregated data were not provided in the report and could not be obtained from the respective directorates of health.

The data on all births and total births by cesarean section were also extracted from the 2008 Annual Report of the Iraqi Ministry of Health [21], which is the earliest published report providing detailed data. The cesarean section rates of all births in 2008 were calculated for each governorate and the rates were compared with the 2012 rates to determine the trend in the cesarean section rate from 2008 to 2012. The relative change between the two time periods was computed for each governorate ((cesarean section rate 2012 – cesarean section rate 2008)/cesarean section rate 2008). The effect of the number of private hospitals in the different governorates on the cesarean section rate was examined using a linear regression. The data were analyzed using SPSS statistical software (version 19). A *P* value of ≤ 0.05 was considered statistically significant.

## Results

Of the 1,300,103 births recorded in Iraq in 2012, 29.6% were home births and 70.4% were hospital-based births, 61.8% of which were in public hospitals and 8.6% in private hospitals. The overall cesarean section rate for all births in Iraq in 2012 was 24.4%. The cesarean section rates did not differ much between the Iraqi Kurdistan Region and the Center/South of Iraq (25.4 and 24.3%, respectively). The rate was specifically high in the governorates of Baghdad (38.9%), Al-Diwaniya (35.1%), Erbil (31.4%) and Al-Najaf (30.1%). The overall hospital-based cesarean section rate was (34.7%). The rate was slightly higher in the Center/South of Iraq compared to the Iraqi Kurdistan Region (34.9 and 33.8%, respectively). The cesarean section rate in public hospitals in the Center/South of Iraq ranged between 17.4 and 38.8%. This rate was the highest in Baghdad (38.8%), followed by Al-Diwaniya (35.7%) and Al-Anbar (33.8%). The cesarean section rate in private hospitals ranged between 35% and 100%. The segregated data for the hospital-based cesarean section rate in the Iraqi Kurdistan Region was not available. The details of the distribution of all births and hospital-based cesarean section rates in the different governorates of Iraq are shown in Table 1. The cesarean section rates were significantly higher in the governorates that had a larger number of private hospitals compared to those with a smaller number or no private hospitals (beta = 0.671; *r* = 0.671; *P* < 0.002).

**Table 1:** Distribution of all births and hospital-based cesarean section rates according to the governorates for the year 2012

Governorates	All births		Public hospitals		Private hospitals		No. of private hospitals
	No. of births	CS rate %	No. of births (%)	CS rate %^a^	No. of births (%)	CS rate %^a^	
Baghdad	254,862	38.9	136,531 (53.6)	38.8	60,679 (23.8)	76.2	36
Basrah	102,386	22.9	83,011 (81.1)	26.2	4798 (4.7)	35.0	4
Nineveh	147,917	15.2	97,951 (66.2)	22.3	680 (0.5)	100.0	3
Maysan	42,566	9.5	23,238 (54.6)	17.4	0 (0.0)	-	0
Al-Diwaniya	41,761	35.1	26,955 (64.5)	35.7	5100 (12.2)	98.6	3
Diyala	61,400	20.1	33,087 (53.9)	32.1	1725 (2.8)	100.0	3
Al-Anbar	58,096	24.0	34,908 (60.1)	33.8	3710 (6.4)	57.5	2
Babylon	64,563	21.0	44,249 (68.5)	26.9	1696 (2.6)	100.0	4
Kerbala	44,693	26.7	30,770 (68.8)	24.4	4925 (11.0)	89.4	2
Kirkuk	55,078	19.0	32,940 (59.8)	28.0	1262 (2.3)	98.4	2
Wasit	58,527	18.5	31,461 (53.8)	34.4	0 (0.0)	-	0
Thi-Qar	66,034	16.6	38,898 (58.9)	23.3	1905 (2.9)	99.8	2
Al-Muthanna	35,461	17.1	25,960 (73.2)	23.4	0 (0.0)	-	0
Salah Al-Deen	55,202	14.0	21,767 (39.4)	26.1	2274 (4.1)	88.9	2
Al-Najaf	50,762	30.1	40,029 (78.9)	31.1	3114 (6.1)	91.2	3
Centre/South	1,139,308	24.3	701,755 (61.6)	29.3	91,868 (8.1)	77.9	66
Erbil	55,864	31.4	37,666 (67.4)	NA	9012 (16.1)	NA	13
Dohouk	52,569	15.9	27,909 (53.1)	NA	5117 (9.7)	NA	3
Al-Sulaimaniya	52,362	27.5	35,626 (68.0)	NA	6118 (11.7)	NA	14
Kurdistan	160,795	25.4	101,201 (62.9)	-	20,247 (12.6)	-	30
Total Iraq	1,300,103	24.4	802,956 (61.8)	-	112,115 (8.6)	-	96

*CS* cesarean section, *NA* data not available

^a^The distribution for the Iraqi Kurdistan Region is not included because the segregated data for the public/private hospitals was unavailable. The cesarean section rates for all hospital births were Erbil: 37.6%, Dohouk: 26.6%, Al-Sulaimaniya: 34.5%, Kurdistan: 33.8%, Centre/South: 34.9%, and total Iraq: 34.7%

The overall rate of cesarean section increased remarkably from 18.0% in 2008 to 24.4% in 2012. The increase was higher in the Iraqi Kurdistan Region compared to the rest of Iraq (from 14.6 to 25.4% versus from 18.4 to 24.3%). The rate increased in all of the governorates during this period except Maysan. The increase was highest for Erbil, Basrah, Al-Sulaimaniya and Kirkuk with a relative change of 116.6, 90.8, 58.0 and 52.0%, respectively (Table 2).

**Table 2:** Change in the cesarean section rates in the different governorates from 2008 to 2012

Governorates	2008		2012		Relative change in %^a^
	No. of births	CS rate %	No. of births	CS rate %	
Baghdad	216,883	28.5	254,862	38.9	36.5%
Basrah	96,137	12.0	102,386	22.9	90.8%
Nineveh	117,967	12.6	147,917	15.2	20.6%
Maysan	39,092	9.5	42,566	9.5	0.0%
Al-Diwaniya	39,022	28.7	41,761	35.1	22.3%
Diyala	42,676	17.0	61,400	20.1	18.2%
Al-Anbar	38,693	17.0	58,096	24.0	41.2%
Babylon	70,488	20.7	64,563	21.0	1.4%
Kerbala	39,331	19.8	44,693	26.7	34.8%
Kirkuk	45,887	12.5	55,078	19.0	52.0%
Wasit	47,513	16.0	58,527	18.5	15.6%
Thi-Qar	66,971	11.3	66,034	16.6	46.9%
Al-Muthanna	31,498	14.7	35,461	17.1	16.3%
Salah Al-Deen	45,093	11.0	55,202	14.0	27.3%
Al-Najaf	46,999	24.2	50,762	30.1	24.4%
Centre/South	984,250	18.4	1,139,308	24.3	32.1%
Erbil	40,667	14.5	55,864	31.4	116.6%
Dohouk	41,364	11.7	52,569	15.9	35.9%
Al-Sulaimaniya	41,935	17.4	52,362	27.5	58.0%
Kurdistan	123,966	14.6	160,795	25.4	74.0%
Total Iraq	1,108,216	18.0	1,300,103	24.4	35.6%

*CS* cesarean section

^a^(Cesarean section rate last time period – cesarean section rate first time period)/cesarean section rate first time period

## Discussion

The overall cesarean section rate in Iraq in 2012, as revealed by this study (24.4%), is far higher than the recommended level of 10% [5]. The Iraq MICS 2011 showed a slightly lower cesarean section rate (22.2%) than that revealed by the present study [20]. The cesarean section rate in Iraq is greater than the rate in some neighboring countries such as Jordan (18.5%) [22], but it is considerably lower than the rate in Iran (40%) [23]. In general, Western Asia, including Iraq, is one of the regions of the world with high cesarean section rates, which are on an average of 26.8%. However, this rate varies widely among the countries of the region ranging from 4.8% in Yemen to 47.5% in Turkey [24].

The rates of cesarean section in the different governorates of Iraq are in line with the findings of the Iraq MICS 2011, which revealed variations in the rates ranging from 16 to 38% with the governorates of Al-Sulaimaniya, Al-Diwaniya, Erbil, Kerbala, Najaf and Baghdad showing the highest rates [20].

The overall hospital-based cesarean section rate in Iraq was considerably high and exceeded the rates of many other Arab countries. A review of the hospital-based cesarean section rates in 18 Arab countries, not including Iraq, reported lower rates ranging from 5.3% in Mauritania (2003) to 26.2% in Egypt (2000) [25]. The high hospital-based cesarean section rate might be indirectly attributed to having many low-risk pregnancies delivered at home, whereas the high-risk pregnancies and the elective or planned cesarean sections are referred to hospitals. Thus, the rate of cesarean sections in hospitals increases proportionally.

The private hospital-based cesarean section rates in most of the governorates in the Center/South of Iraq were very high. It is expected that these rates would be higher in the Iraqi Kurdistan Region; however, these segregated data are unfortunately not available. The very high cesarean section rate in private hospitals is the main contributor to the high hospital-based cesarean section rate in Iraq. Private hospitals in Iraq are primarily surgical hospitals that provide cesarean section services, and many of these facilities do not provide normal vaginal delivery services. This could be the reason why some of the governorates showed private hospital-based cesarean section rates of up to 100%.

The trend of an increasing cesarean section rate is not limited to Iraq because most countries follow a similar or a more rapidly rising trend. The global average cesarean section rate increased from 6.7% in 1990 to 19.1% in 2014 with an average annual rate of increase of 4.4%. The increasing trend was remarkable in Western Asia where the cesarean section rate increased from 6.3 to 28.1% with an average annual rate of increase of 6.4% [24].

The recent global increase in the cesarean section rate can be largely attributed to different factors including advances in technologies to detect fetal distress and the attitudes of clinicians and women toward cesarean sections such as the avoidance of labor pain [26]. In Iraq, there is a lack of clear evidence of the main factors attributing to the reported high cesarean section rate. However, a previous study showed that 11% of cesarean sections in a public hospital in Baghdad were conducted for non-medical reasons such as the fear of vaginal delivery (45.7%), choosing the right timing for security reasons (31.4%), avoiding delivery pain (14.3%) and performing a tubal ligation (8.6%) [12].

Another important potential factor for the high cesarean section rate is provider-induced demand because there is clear evidence of an association between private care and an increased rate of cesarean sections [26]. This factor could be relevant to the Iraqi context since governorates with a larger number of private hospitals had increased rates of cesarean sections. This factor could also explain the larger increase in the cesarean section rate in the Kurdistan Region because this region has recently witnessed a rapid expansion of a poorly regulated private healthcare sector [14]. Iraq may potentially be prone to the problem of provider-induced demand due to a lack of consumer organizations and patients’ rights groups to protect patients’ interests and the absence of health insurance agencies to scrutinize providers and prevent the easy abuse of these services. In addition, Iraq relies on the fee-for-service or out-of-pocket payment for private healthcare services, which increases the risk of provider-induced demand [8, 15].

The present study only assessed the cesarean section rate, which, alone, is not a proper indicator to monitor the quality of emergency obstetric care. Heemelaar et al. [27] conducted a criteria-based audit of cesarean sections by measuring the prevalence of substandard quality of care that led to unnecessary cesarean sections and a delay in performing interventions to prevent cesarean sections. They identified high rates of unnecessary and potentially preventable cesarean sections. Another study also showed that a clinical audit is an effective strategy for reducing the cesarean section rate and recommended strict monitoring of the indications for cesarean sections to reduce the cesarean section rate [28]. Therefore, future research needs to consider conducting an audit of the indications for cesarean sections rather than measuring the cesarean section rate alone.

This study is limited by being a descriptive study based on already available data. This study provides information about the rates and trends of cesarean sections in Iraq, but does not look at the actual reasons for the increasing trend. It does not show whether this trend is related to medical reasons or non-medical reasons such as the women’s preferences or provider-induced demand. This study is based on data extracted from the annual reports of the Ministry of Health. These reports are primarily dependent on the completeness of birth registration and the accurate reporting of births by the civil registration offices and the directorates of health at the governorate level. According to UNICEF, birth registration is nearly universal in Iraq [19, 20]. However, the issue of accuracy of reporting birth records remains a concern, which adds another limitation to this study.

## Conclusions

The overall cesarean section rate in Iraq of 24.4% is far above the recommended rate of 10%. Iraq has witnessed a rapid upward trend in the cesarean section rate from 2008 to 2012, with much of this trend being attributed to the Kurdistan Region. The hospital-based rate of cesarean sections, particularly in private hospitals, is extremely high. There is a potential relationship between the expansion of the private health sector and the increasing rate of cesarean sections, particularly in the Kurdistan Region. Further research is needed to monitor the indications for cesarean sections and explore the determinants of this rapid upward trend of cesarean sections in Iraq.

## Abbreviations

CS: Cesarean section
MICS: Multiple Indicator Cluster Survey
